# Fatty acids and neuropsychiatric disorders

**DOI:** 10.1186/1471-244X-8-S1-S1

**Published:** 2008-04-17

**Authors:** Basant K Puri

**Affiliations:** 1 MRI Unit, MRC Clinical Sciences Centre, Imaging Sciences Department, Imperial College London, Hammersmith Hospital, Du Cane Road, London W12 0HS, UK

In 1937, after qualifying as a medical doctor and before taking up his appointment as a fellow at Magdalen College, Oxford, Hugh Sinclair visited Herbert Evans, the co-discoverer with Katherine Scott Bishop of vitamin E, and became interested in the possibility that some fatty acid deficiencies might account for the rise in ischaemic heart disease in the Western world [1,2]. In his research letter entitled 'Deficiency of essential fatty acids and atherosclerosis, etcetera' published in the *Lancet *in 1956 [3], Sinclair provided evidence that cardiovascular disease and, in particular, atherosclerosis, might be associated with a deficiency of what we now call long-chain polyunsaturated fatty acids; he suggested that the etcetera pointed to the likelihood that diseases other than cardiovascular disease might be related to a deficiency in fatty acids [2,3]. One of his students at Oxford, David Horrobin, was instrumental in establishing a theoretical framework demonstrating that this might be true of neuropsychiatric disorders [4]. In turn, it was my very great privilege and honour to collaborate with, and be taught by, the late David Horrobin while establishing that fatty acid interventions might indeed have a therapeutic part to play in neuropsychiatric disorders ranging from depression to Huntington's disease (chorea) [5,6].

In December 2007 the Organizing and Scientific Committee of the 3^rd ^International Congress on Brain & Behaviour invited me to chair a session on the theme of fatty acids and neuropsychiatric disorders. This supplement is based on the proceedings of that meeting and includes papers co-authored by Dr. Marcelo Bustos, Professor Graeme Bydder, Professor Massimo Cocchi, Dr. Serena Counsell, Dr. Gavin Hamilton, Professor Brian Ross, Dr. Nadeem Saeed, Dr. Lucio Tonello, Dr. Ian Treasaden, Dr. Sofia Tsaluchidu and me. In the first paper [7], evidence is provided from the *in vivo *31-phosphorus neurospectroscopy phosphodiester peak which indexes the phosphorylated polar head groups glycerophosphorylcholine and glycerophosphorylethanolamine that exhaled ethane is a biomarker of cerebral *n*-3 polyunsaturated fatty acid peroxidation in humans, a finding which clearly may have implications for the use of this non-invasive biomarker in future research. The second paper [8], entitled 'The use of artificial neural networks to study fatty acids in neuropsychiatric disorders' demonstrates for the first time that the self-organizing map, an unsupervised competitive-learning network algorithm which forms a nonlinear projection of a high-dimensional data manifold on a regular, low-dimensional grid, is an optimal type of artificial neural network to use for the task of analyzing platelet fatty acids in neuropsychiatric disorders. Cigarette smoking appears to be relatively common in sufferers of certain neuropsychiatric disorders, such as schizophrenia. It is important, therefore, to ascertain the extent to which smoking is a confounder when studying oxidative stress in such disorders. This is the subject of the third paper [9], 'A comparison of oxidative stress in smokers and non-smokers: an *in vivo *human quantitative study of *n*-3 lipid peroxidation', which has a surprising result. The fourth paper [10], 'Fatty acids and oxidative stress in psychiatric disorders', is a systematic review of published evidence for increased oxidative stress in such disorders. The fifth paper [11] presents the first voxel-based morphometry study of grey matter changes in the whole brain in schizophrenia associated with a history of seriously and violently offending. Finally, the last paper [12] describes cerebral spectroscopic and oxidative stress studies in patients with schizophrenia who have dangerously violently offended.

I should like to express my thanks to the organizers of the conference and to the editor and staff of *BMC Psychiatry*, with particular thanks to Ros Dignon of BioMed Central, who has been of immense help in putting this supplement together.
